# PSF functions as a repressor of hypoxia-induced angiogenesis by promoting mitochondrial function

**DOI:** 10.1186/s12964-020-00684-w

**Published:** 2021-02-11

**Authors:** Lijie Dong, Wenbo Li, Tingting Lin, Boshi Liu, Yaru Hong, Xiaomin Zhang, Xiaorong Li

**Affiliations:** 1 Tianjin Key Laboratory of Retinal Functions and Diseases, Tianjin, People’s Republic of China; 2 Tianjin International Joint Research and Development Centre of Ophthalmology and Vision Science, Tianjin, People’s Republic of China; 3 Eye Institute and School of Optometry, Tianjin Medical University Eye Hospital, 251 Fukang Road, Nankai, Tianjin, 300384 People’s Republic of China

**Keywords:** PSF, Hakai, VEGF, Neovascularization, HIF1-α, Hypoxia, Mitochondrion

## Abstract

**Background:**

Abnormal neovascularization is the most common cause of blindness, and hypoxia alters tissue metabolism, function, and morphology. HIF-1α, the transcriptional activator of VEGF, has intricate mechanisms of nuclear translocation and activation, but its signal termination mechanisms remain unclear.

**Methods:**

We investigated the role of polypyrimidine tract-binding protein-associated splicing factor (PSF) in cellular energy production, migration, and proliferation by targeting HIF-1α in vivo and in vitro PSF plasmids were transfected with liposome 2000 transfection reagent. Young C57/BL6J mice were kept in a hyperoxia environment, followed by indoor air, resulting in oxygen-induced retinopathy. Oxygen-induced retinopathy (OIR) animals were randomly divided into three groups: OIR group, OIR + vector group (OIR cubs treated with rAAV vector) and OIR + PSF group (OIR cubs treated with rAAV-PSF). Age-matched C57/BL6J mice were used as controls and exposed to constant normoxic conditions. The animals were executed and their pupils were subjected to subsequent experiments. The metabolic spectrum was analyzed by Seahorse XFe96 flux analyzer, and OCR and extracellular acidification rate were quantified at the same time.

**Results:**

PSF ameliorated retinal neovascularization and corrected abnormal VEGF expression in mice with oxygen-induced retinopathy and reduced intra-retinal neovascularization in Vldlr − / − mice. PSF reprogrammed mitochondrial bioenergetics and inhibited the transition of endothelial cells after hypoxia, suggesting its involvement in pathological angiogenesis.Ectopic PSF expression inhibited hypoxia-induced HIF-1α activation in the nucleus by recruiting Hakai to the PSF/HIF-1α complex, causing HIF-1α inhibition. PSF knockdown increased hypoxia-stimulated HIF-1α reactions. These hypoxia-dependent processes may play a vital role in cell metabolism, migration, and proliferation. Thus, PSF is a potential treatment target in neovascularization-associated ophthalmopathy.

**Conclusion:**

This is the first study showing that PSF inhibits HIF-1α via recruitment of Hakai, modulates mitochondrial oxidation and glycolysis, and downregulates VEGF expression under hypoxia. We propose a new HIF-1 α/Hakai regulatory mechanism that may play a vital role in the pathogenesis of neovascularization in ophthalmopathy.

PSF-Hakai–HIF-1α signaling pathway under hypoxia condition. Schematic diagram showing that the PSF-Hakai–HIF-1α signaling pathway. Under hypoxia condition, PSF-Hakai complex regulate HIF-1α signaling, thus inhibiting downstream target gene VEGF, cell metabolism and angiogenesis eventually.
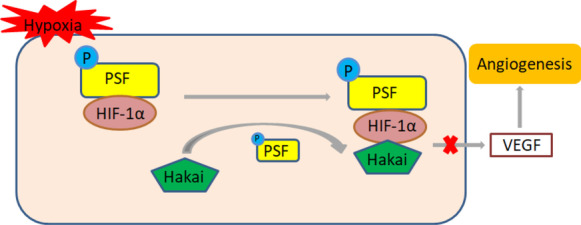

**Video Abstract:** Detailed information of Materials and Methods.

## Background

Hypoxia can lead to a series of pathological processes and cause anomalous changes in tissues and organs with respect to metabolism, function, and morphological structure [1–3]; As an upstream mediator of vascular endothelial growth factor (VEGF), hypoxia-inducible factor-1 alpha (HIF-1α) is a significant transcription factor for cellular response in hypoxia, and also promotes angiogenesis and boosts metabolism, thus provoking abnormal vascularization [4–6]. However, the fate of this protein is mainly determined by the O2/PHD/pVHL pathway, which is responsible for its ubiquitination and degradation [7, 8].

Recently, our group focused on the potential effect of PSF on pathological ocular angiogenesis. We found that PSF is a transcriptional inhibitor that prevents the activation of immunoglobulin (Ig) gene transcription mediated by signal transducer and activator of transcription-6 through histone deacetylase recruitment [9]. Moreover, our data further confirmed that PSF is a suppressor of IGF-1-induced VEGF gene transcription by recruiting Hakai [10]. The E3 ubiquitin ligase Hakai is the first reported post-translational regulator of E-cadherin complex. Hakai specifically targets the degradation of E-cadherin, thereby reducing the contact between epithelial cells and cells [11]. Studies have shown that Hakai plays an important role in various cellular processes [12] and tumorigenesis [13]. According to reports, Hakai is also involved in the control of cell proliferation [11] and promotes the expression of cancer-related genes [14]. The overexpression of Hakai not only affects the contact between cells, but also affects the proliferation of epithelial cells and fibroblasts by reducing cell matrix adhesion [13] and enhancing cell invasiveness [15]. PSF is an RNA-binding protein and a new Hakai-interacting protein [16]. Studies have shown that PSF short hairpin RNA or dominant negative PSF mutants can significantly inhibit the proliferation of Hakai overexpressing cells [14]. Hakai can affect cell proliferation and oncogene phenotype by improving the ability of PSF to bind RNA, thereby promoting cancer-related genes expression [16]. In addition, knocking out PSF can inhibit Hakai-induced cell proliferation [14]. So far, no evidence has been found that Hakai can induce PSF ubiquitination. Therefore, there may be unrecognized substrate proteins in Hakai/PSF-mediated proliferation regulation. Moreover, it has been found that nuclear translocation of HIF-1α directly or indirectly regulates mitochondrial function [17]. However, previous studies have seldom reported the termination mechanism of HIF-1α cascade reaction in the nucleus after hypoxic preconditioning. Therefore, we hypothesized that the PSF-Hakai complex may have beneficial effects on neovascularization via HIF-1α inhibition.

The purpose of this study was to identify the role of PSF in cellular metabolic regulation in human retinal microcapillary endothelial cells (HRMECs) reflected by their proliferation and migration. We targeted HIF-1α to investigate the role of PSF in cell energy production, proliferation, and migration. Our results emphasize the relationship between the PSF-Hakai–HIF-1α signaling pathways, mitochondrial function, HRMEC proliferation and migration related to pathologic neovascularization.

## Results

### PSF improved retinal neovascularization (RNV) and rectified abnormal VEGF production in an oxygen-induced retinopathy (OIR) model

**Fig. 1:**
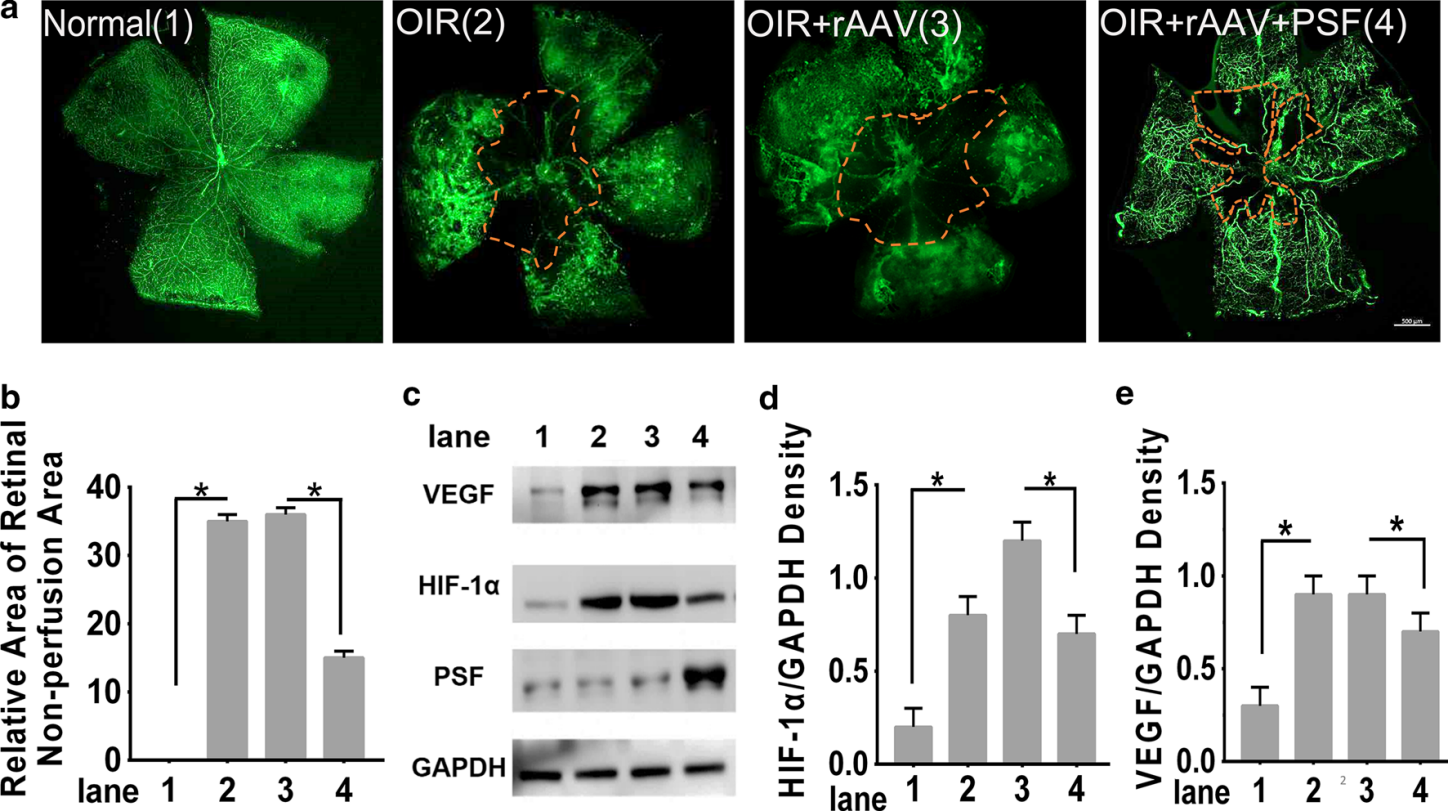
rAAV-PSF intravitreal injection normalized the retinal vasculature and VEGF production in an OIR model. **a** Flat-mounted retinas were obtained to track changes with or without PSF administration. Avascular areas are highlighted with a red outline. **b** Quantification analysis of avascular areas. **c** Retinal VEGF and HIF-1α shown by Western blotting, n = 6/group. **d** Grey density of VEGF (D) and HIF-1α (E) in the different groups

To examine the importance of PSF in the RNV process, we used the well-established OIR mouse model to imitate pathologic development of RNV and treated them with recombinant adeno-associated virus (rAAV) or rAAV-PSF particles intravitreally on postnatal (P) day 12 and P15. Retinal flat-mounts were used to visualize the retinal vasculature and the avascular area. The levels of VEGF, HIF-1α and PSF were estimated by western blotting on P17. As shown by the retinal flat-mounts (Fig. 1a), rAAV-PSF rather than rAAV, administration effectively reduced the avascular areas to 53% compared to that in OIR counterparts (Fig. 1b), which indicating that PSF restrained retinal neovascularization. To identify the mechanisms underlying these effects of PSF, we further compared the levels of VEGF and HIF-1α In OIR retinas, the expression of VEGF and HIF-1α increased compared to that in normal controls (Fig. 1c, d, e). However, rAAV-PSF markedly inhibited HIF-1α and VEGF (Fig. 1c, d, e). These results suggested that PSF downregulated the hypoxia-induced VEGF increase in the OIR retina.

### PSF reduced retinal vascular leakage and decreased intra-retinal neovascularization (IRNV) in Vldlr − / − mice

Very-low-density lipoprotein receptor (VLDLR) in knockout mice (vldlr(− / −)) has been reported to induce subretinal neovascularization. The retinal pathogenic process in vldlr(− / −) mice recapitulates key features of retinal angiomatous proliferation (RAP) in humans, a subtype of neovascular age-related macular degeneration. The vldlr(− / −) mouse exhibits histologic and angiographic characteristics of RAP and is a reproducible animal model facilitating studies of the molecular mechanisms of RAP [18].

**Fig. 2:**
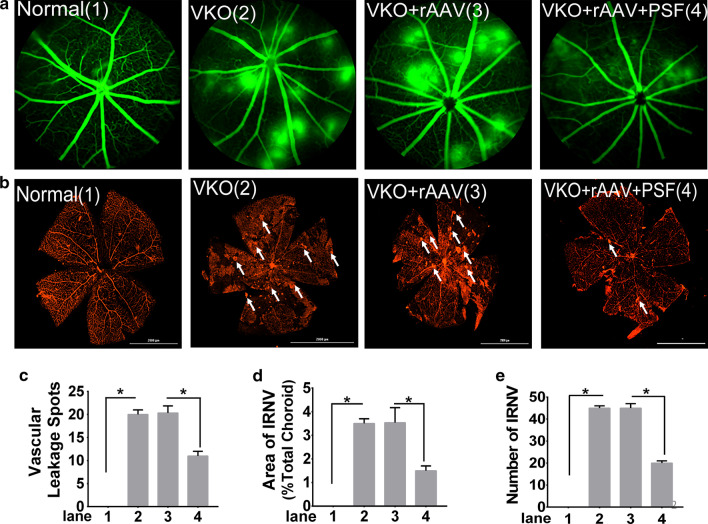
PSF reduced retinal vascular leakage and ameliorated IRNV in Vldlr − / − mice. rAAV-PSF or rAAV vechile intravitreal injection were performed in Vldlr − / at P17.FFA was used as a tracer to track retinal vascular leakage. **a** A representative image of retinal vascular leakage, **b** flat mount of retina visualized by IB4 staining with white arrows indicating neovascularization, **c** quantification of the number of leakage spots, **d** IRNV areas in total retian, **e** statistic analysis of IRNV number

The influence of PSF on retinal vascular leakage were observed by fundus fluorescein angiography (FFA). A large number of intense hyper fluorescent spots (Fig. 2a) were observed in Vldlr − / − retina, indicating dye leakage, whereas less retinal leakage spots were observed in the PSF treatment group (Fig. 2b), which is confirmed by statistical analyses (Fig. 2c). We further assessed the influence of PSF on the development of IRNV in Vldlr − / − mice. As shown by IB4 stained flat-mounts of retina (Fig. 2b)**,** the number of IRNV were 40.26 ± 3.04 in vehicle-treated Vldlr − / − mice (n = 6), and reduced to 10.38 ± 2.09 in the PSF treatment group (n = 6), representing an approximate 75% reduction in IRNV (Fig. 2d), while the areas of IRNV were also markedly reduced in PSF-treated Vldlr − / − mice (Fig. 2e). The data showed that PSF inhibited the formation of IRNV in Vldlr − / − mice.

### PSF inhibited hypoxia-induced angiogenesis of HRMECs

**Fig. 3:**
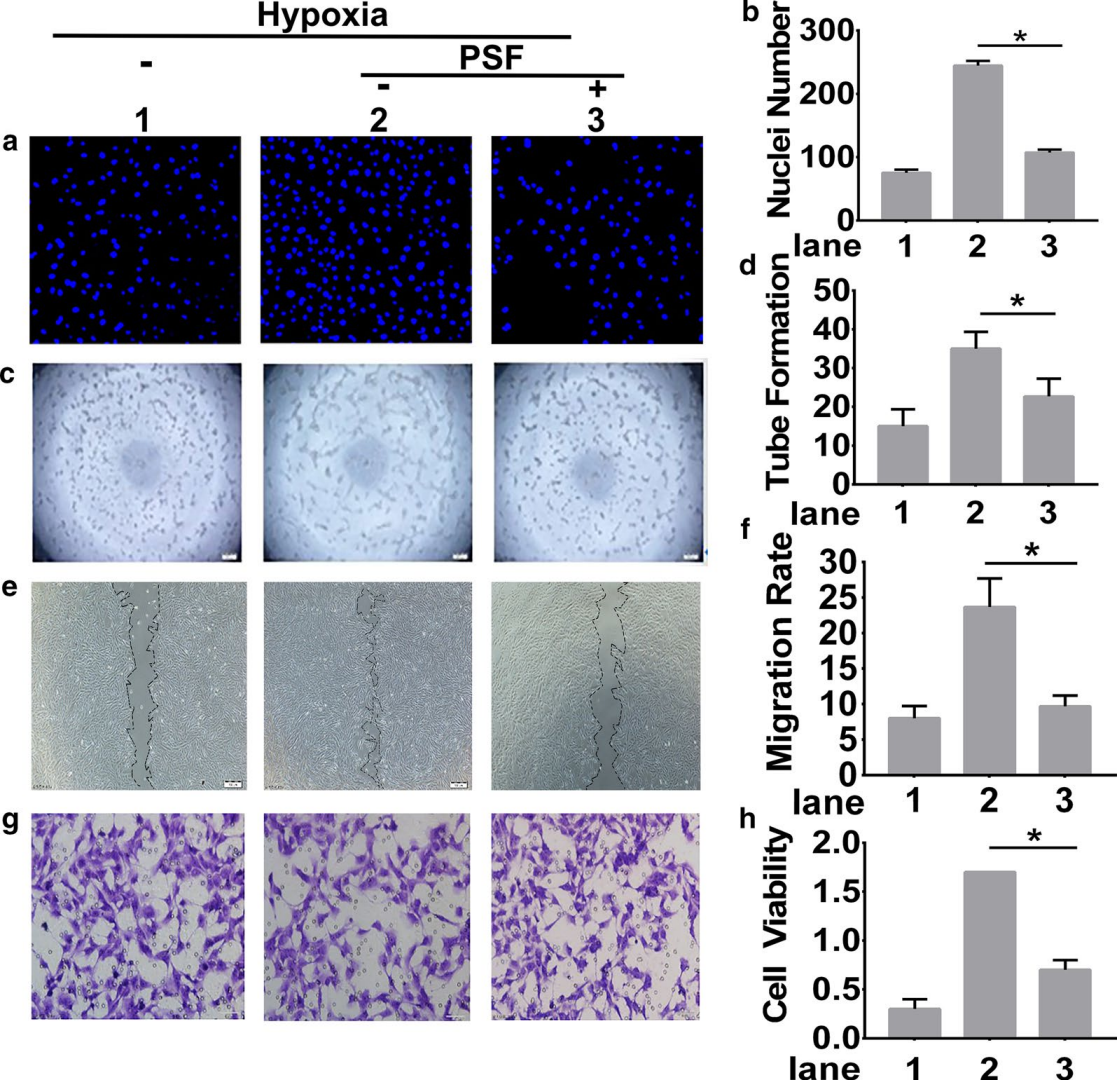
PSF was implicated in hypoxia-induced angiogenesis of HRMECs. HRMECs were pretreated or untreated with PSF and then placed in a hypoxic environment. Proliferatiion was analyzed using Hoechst staining (**a**) and combined with statistic quantificaition (**b**), Tube formation and branching points were determined (**c**) and quantified (**d**), Wound healing assays were determined (**e**) and quantified (**f**), Transwell assays (**g**) and combined with statistic quantificaition (**h**) were used to determine the number of migrated cells

To further explore the involvement of PSF in angiogenesis, we performed proliferation, tube formation, wound healing assay, and Transwell assay to simulate various aspects of angiogenesis. Higher numbers of proliferating cells (Fig. 3a, b), tube formation (Fig. 3c, d) and migrating cells (Fig. 3e, f) in HRMECs were detected after hypoxia induction compared to that in PSF treatment group. Quantitative analysis of the results indicated that PSF dramatically reduced the rates of proliferation (Fig. 3b), tube formation (Fig. 3d) and migration (Fig. 3g, h), compared to that in the vehicle control. These data indicated that PSF blocked hypoxia-induced angiogenesis in HRMECs.

### PSF affected the mitochondrial bioenergetic and glycolytic profiles under hypoxic condition

**Fig. 4:**
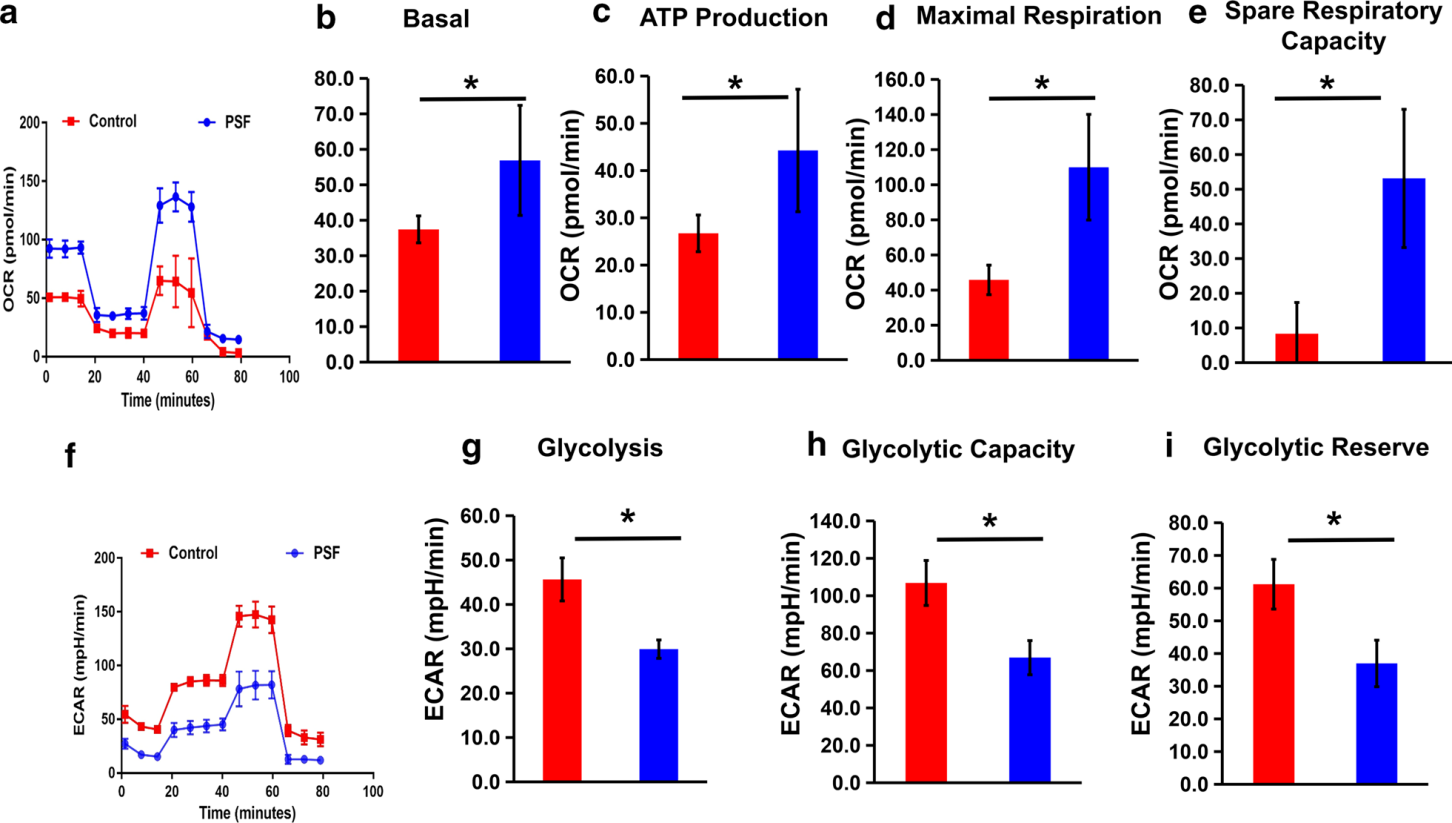
PSF affected the mitochondrial bioenergetic and glycolytic profiles under hypoxic conditions. After exposure to hypoxia, the mitochondrial respiration and glycolytic functions in PSF-treated or vehicle HRMECs were measured using Seahorse analysis. **a** Representative traces identifying the pattern of OCR in mitochondria stress tests. **b** Basal OCR, **c** ATP production, **d** maximal respiration and **e** spare respiratory capacity in ectopic PSF cells. **f** The indicative curve for the average extracellular acidification rate (ECAR) within glycolysis stress assays, **g** glycolysis, **h** glycolytic capacity, and **i** the reservation of glycolysis were indicated.*P < 0.05

Mitochondrial oxidation and glycolysis coordination have an influence on the fate of cells. We studied the function of PSF in regulating the mitochondrial glycolytic profile in HRMECs. After cells were transfected with PSF, the mitochondria respiration and glycolytic function were determined by OCR (Fig. 4a) and ECAR (Fig. 4f) profile. Mitochondria-related index, including basal OCR (Fig. 4b), ATP generation (Fig. 4c), and maximal (Fig. 4d) and spare respiration capacity (Fig. 4e) increased, whereas glycolysis (Fig. 4g), glycolytic capacity (Fig. 4h) and glycolytic reserve (Fig. 4i) reduced after ectopic PSF treatment in HRMECs exposed to hypoxic conditions. Taken together, these metabolic profiles indicated that PSF positively regulated mitochondrial oxidation but negatively regulated glycolysis in HRMECs.

### PSF Interacted with HIF-1α in a hypoxia-dependent manner

**Fig. 5:**
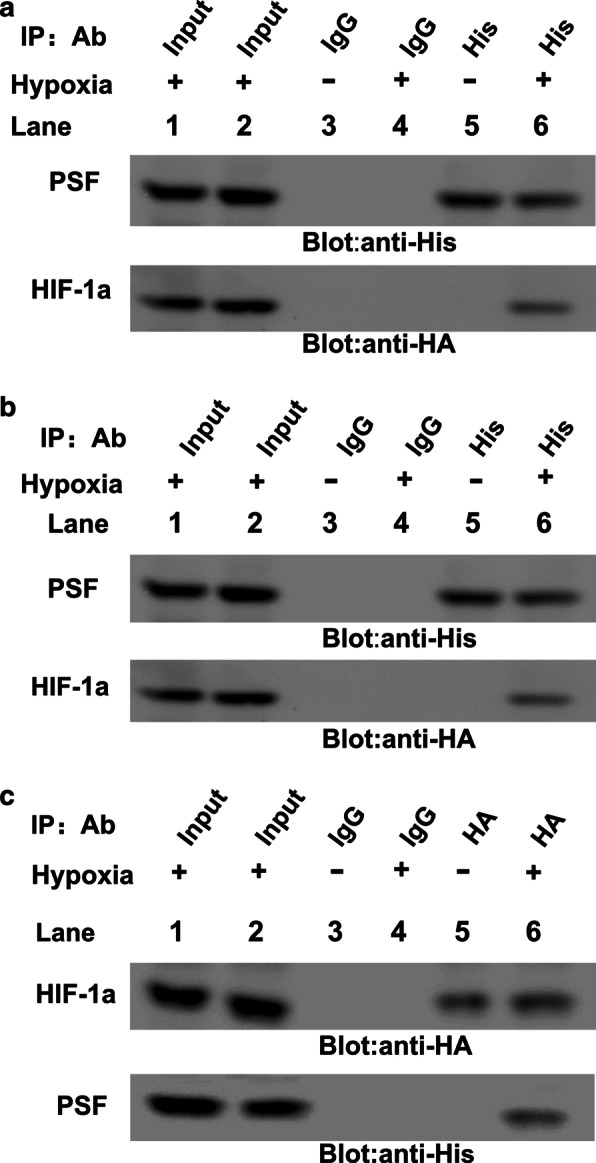
PSF-HIF-1α complex formation under hypoxia induction. Ectopically expressed PSF interacted with HIF-1α were checked using anti-HA antibody in HRMECs as indicated (**a**) and anti-His antibody sedimentation (**b**). **c** Anti-HIF-1αor anti-PSF antibody-based immunoprecipitation with total cell lysates of HRMECs under hypoxia exposure was performed, followed by immunoblotting with the corresponding antibodies as indicated

To analyze whether PSF-HIF-1α complex formation could be seized in vitro, the binding between PSF and HIF-1α was inspected by co-immunoprecipitation assays. Recombinant constructs encoding HA-tagged HIF-1α (HIF-1α–HA) and His-tagged PSF (PSF–His) were co-transfected into HRMECs followed by exposure to hypoxic conditions. Ectopic PSF was purified with anti-His antibodies, and HIF-1α was identified using anti-HA immunoblotting. Co-precipitation of PSF-HIF-1α was detected only after hypoxia stimulation (Fig. 5a, lower panel, lane6). Comparatively, anti-IgG antibody was unable to perform this function. Meanwhile, co-precipitation of HIF-1α and PSF was observed in anti-HA immunoprecipitation (Fig. 5b, lower panel, lane6). These results showed that ectopic expression of PSF and HIF-1α formed a complex after hypoxia. Moreover, endogenous formation of a complex between PSF and HIF-1α was also tested in HRMECs. After exposure to hypoxia, the cells were lysed and the total cell lysates were immunoprecipitated with anti-PSF antibody; rabbit anti-IgG was used as a control. The extracts were then reacted with anti-PSF or anti-HIF-1α antibodies. Consistent with previous results, PSF-HIF-1α binding was detected in hypoxia-treated samples (Fig. 5c, lower panel, lane6). These results further verified that PSF and HIF-1α bind in a hypoxia-dependent manner in vitro.

### Hypoxia promoted co-localization of PSF and HIF-1α as well as enhanced tyrosine phosphorylation of PSF in a time-dependent manner

**Fig. 6:**
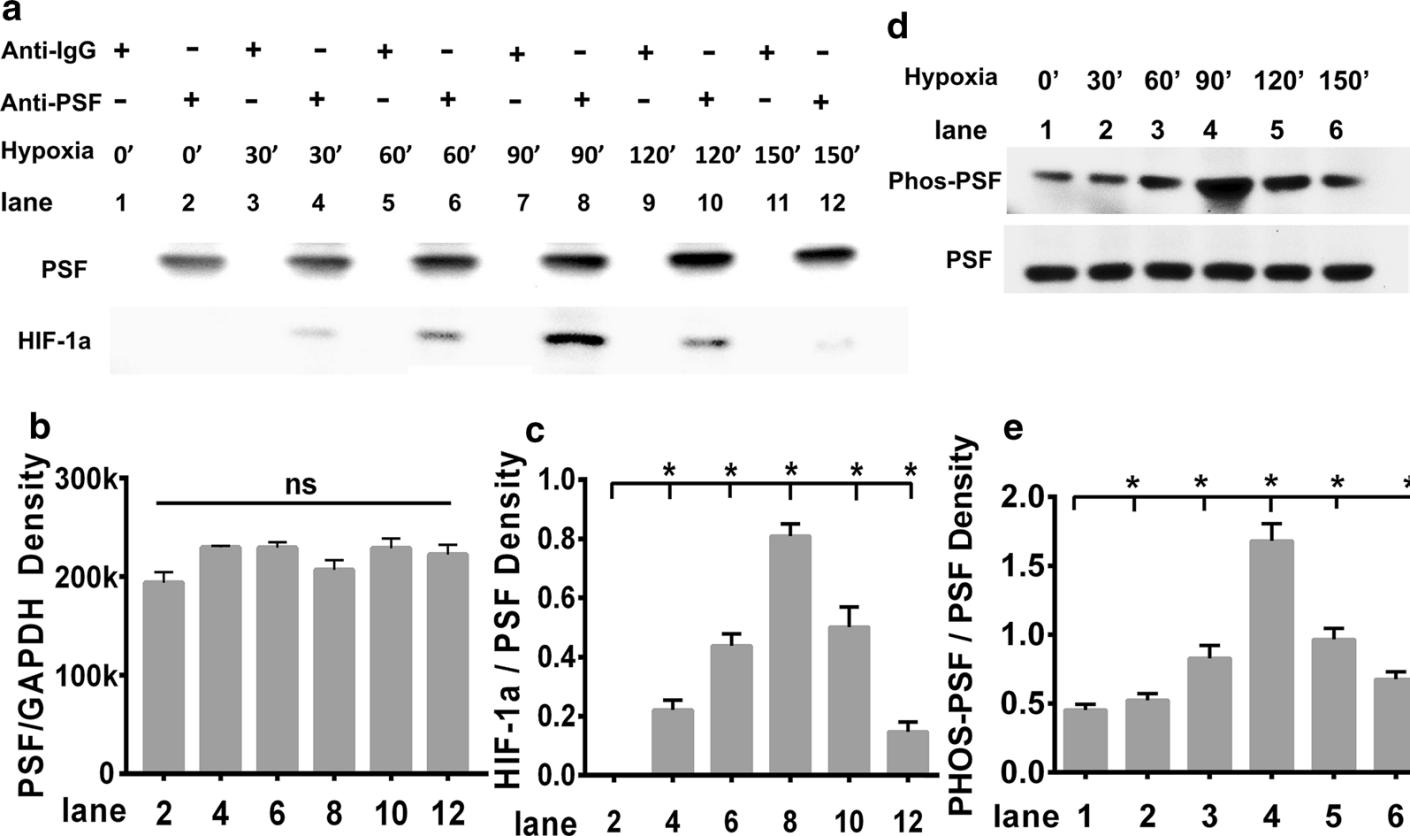
Hypoxia promoted co-localization of PSF and HIF-1α as well as enhanced tyrosine phosphorylation of PSF in a time-dependent manner. **a** Binding of PSF and HIF-1α at various time points under hypoxia condition. **b** Quantitative analysis of PSF at different time points under hypoxic conditions. **c** The amount of bound HIF-1α to PSF. **d** The levels of tyrosine phosphorylated PSF in HRMEC total cell lysates. **e** Phosphorylated PSF was normalized to total PSF

We further explored the potential correlation between PSF-HIF-1α interactions during hypoxia exposure. HRMECs were treated with hypoxia for different time periods as indicated; anti-PSF antibody or rabbit anti-IgG (control) were introduced to pull down the PSF protein complex originating from the total cell lysates, and then reacted with anti-HIF-1α or anti-PSF antibodies. Hypoxia exposure does not affect the basal PSF level, which has been confirmed by blotting (Fig. 6a, upper panel: lanes 2, 4, 6, 8, 10, 12) and quantitative analysis (Fig. 6b), whereas PSF and HIF-1α physical complex formation began 30′ after hypoxia stimulation (Fig. 6a, lower panel: lane 4), and continued for 150 ‘ (Fig. 6a, lower panel: lanes 6, 8, 10, 12); in addition, the amount of complex formed gradually increased following hypoxia treatment. The combination amount of the two partners reaches the maximum value after 90 min intervention; the quantity of precipitated HIF-1α was almost fourfold higher than that of the vehicle group (Fig. 6c). We also assayed the phosphorylation of tyrosine residues in PSF in diverse time points following hypoxia exposure. In the absence of hypoxic stimulation, the tyrosine phosphorylation levels of PSF were at the basic level, and the phosphorylation of PSF gradually increased after hypoxia treatment (Fig. 6c). After hypoxia treatment for 90 min, the amount of phosphorylated PSF was threefold greater than that in the vehicle group (Fig. 6d). Thus, these results emphasized that the hypoxia-induced phosphorylation of PSF was a prerequisite for the association of PSF and HIF-1α.

### Hypoxia stimulation promoted the binding of PSF with Hakai

**Fig. 7:**
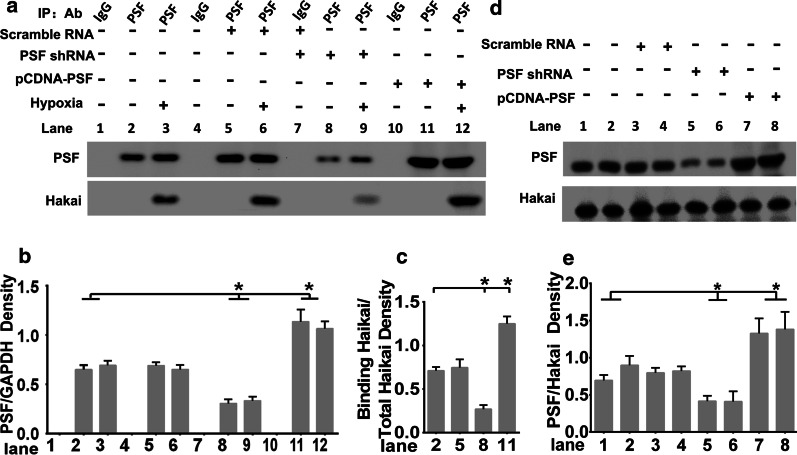
Hypoxia stimulation promoted the binding of PSF with Hakai. **a** Anti-PSF and anti-Hakai immunoblotting in PSF immunoprecipitation complex. **b** The precipitated PSF and **c** the bound Hakai in different treated groups were analyzed by grey density, **d** PSF and Hakai level in total cell lysates were detected by western blot assays. **e** The quantitative analysis of the amounts of PSF protein

To confirm the presence of the PSF-Hakai complex, PSF overexpression and knockdown plasmids were transfected into HRMECs as indicated. As shown in Fig. 7a, pcDNA-His-PSF and pGenesil-PSF-siRNA constructs were able to achieve PSF overexpression (Fig. 7a, upper panel: lanes 11 and 12) and knockdown (Fig. 7a, upper panel: lanes 8 and 9), respectively in HRMECs. Importantly, co-precipitation of the PSF-Hakai complex formed only after hypoxia stimulation. Comparatively, anti-IgG antibody unable to perform this function (Fig. 7a, lower panel: lanes 1, 4, 7, 10). In hypoxia-stimulated samples, ectopic expression of PSF had.

higher potential for binding with Hakai (Fig. 7a, ower panel, lane 11), whereas knockdown of PSF reduced its binding to Hakai (Fig. 7a, lower panel, lane 8). There was no significant difference between the control samples and siRNA scrambled samples (Fig. 7a, lanes 2 and 5). The quantitative analysis of the amounts of PSF protein and Hakai level were performed by Fig. 7b, c. Also the protein level of PSF (upper panel) and Hakai (lower panel) in total cell lysates among different groups were shown by Fig. 7d and grey scale analysis were shown with Fig. 7e, which showing that PSF constructs modulate the PSF level successfully whereas failed to affect Hakai level meantime. The whole figure shows that hypoxia is a prerequisite for the complex formation of PSF and Hakai.

### PSF recruited Hakai to the HIF-1α–PSF complex to inhibit HIF-1α

**Fig. 8:**
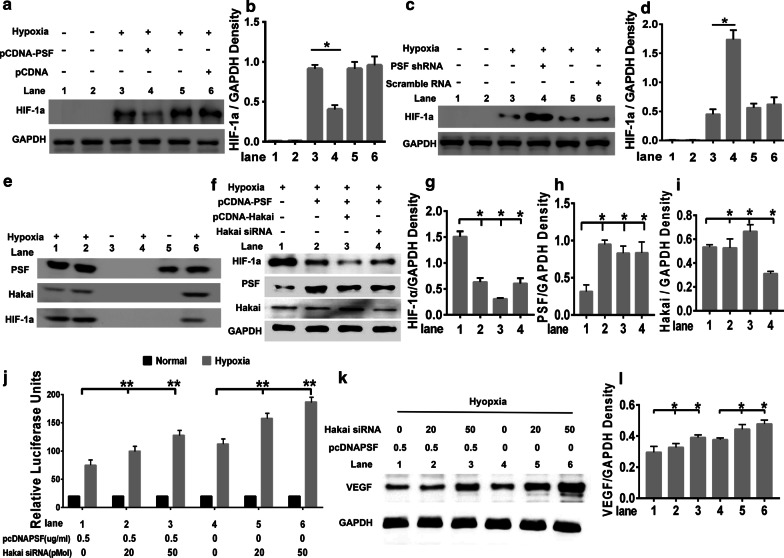
PSF recruited Hakai to inhibit HIF-1α. PSF regulated HIF-1α activation after hypoxia stimulation by Hakai recuitment. Western blotting analysis combined with quantification analysis indicated that PSF overexpression (**a**, b) reduced whereas PSF knockdown (**c**, **d**) the promoted HIF-1α activation levels in nuclear. PSF-Hakai- HIF-1α protein complex formed only after hypoxia induction (**e**). Hakai siRNA reversed the effects of PSF were confirmed by immunoblotting (**f**) and statistic analysis (**g**–**i**), In PSF-treated lysates of HRMEC transfected with different amounts of Hakai protein, VEGF promoter luciferase activity (**j**), VEGF protein level (**k**) and grayscale analysis of VEGF expression were measured (**l**)

To verify the PSF-mediated inhibition of HIF-1α activation through Hakai, we analyzed the nuclear HIF-1α levels using western blotting. Ectopic expression of PSF lowered hypoxia-induced expression of nuclear HIF-1α compared with that in parental cells (Fig. 8a, b, upper panel: lane 4 and 6). Moreover, PSF knockdown enhanced hypoxia-induced expression of nuclear HIF-1α compared with that in parental cells (Fig. 8c, d, upper panel: lane 4 and 6). Collectively, these results demonstrated that PSF acted as an inhibitor of HIF-1α activation when reacting to hypoxia stimulation.

To elucidate the potential relationship among PSF, Hakai, and HIF-1α, co-immunoprecipitation experiments were performed; the ability of PSF to act as a bridge between Hakai and HIF-1α was examined. Antibodies against PSF protein or control antibody were incubated with nuclear cell lysates from control or hypoxia-treated HRMECs, and the sediments were subjected to blotting with indicated antibodies. As shown in Fig. 8e, HIF-1α (lower panel, lane 6) and Hakai (middle panel, lane 6) were detected in the PSF precipitate (upper panel, lane 6) in hypoxia-treated cell lysates, but not in that of the controls (lane 5). To better address the role of Hakai in the PSF-HIF-1α complex, HRMECs were transfected with pcDNA3.1–His–PSF combined with pcDNA3.1–Hakai or Hakai siRNA, and the HIF-1α activation level was compared in these cells. PSF-induced inactivation of HIF-1α in HRMECs (Fig. 8f, upper panel, lane 2) and Hakai (Fig. 8f, upper panel, lane 3) played a synergistic role, whereas Hakai siRNA (Fig. 8f, upper panel, lane 4) played an antagonistic role in PSF action. The grey scale analysis of HIF-1α in each group were shown in Fig. 8g. Meanwhile, the immunoblotting and quantitative analysis results of PSF (Fig. 8f, middle panel) and Hakai (Fig. 8f, lower panel) in each experimental group are shown in the Fig. 8h, i

These data indicated PSF recruited Hakai to the PSF/HIF-1α transcriptional complex under hypoxic conditions.

VEGF is the direct downstream target of HIF-1α transcriptional activation, therefore, to elucidate the outcome of the PSF-Hakai interaction, luciferase activity was introduced in HRMECs. As shown in Fig. 8j, Hakai did not change the basic reporter activity without stimulation. The inhibition of PSF on hypoxia-induced VEGF gene transcriptional activation was reversed by Hakai knockdown treatment (Fig. 8j). Hakai knockdown was incapable of affecting VEGF transcriptional activity without hypoxia stimulation. Importantly, luciferase assays supported the previous result that the inhibitory effects of PSF on hypoxia-stimulated transcription could be restored in a dose-dependent manner through Hakai siRNA processing.

In the PSF-treated lysates of HRMEC transfected with different amounts of Hakai protein, VEGF protein levels (Fig. 8k) were detected and the corresponding gray-scale analysis (Fig. 8l)of VEGF expression was performed. The results of Western blotting are consistent with the results of the reporter gene, and both indicate that the knockdown of Hakia can effectively antagonize the inhibitory effect of PSF on VEGF expression.

Therefore, we conclude that PSF inhibits HIF1-α-induced VEGF gene transcription by recruiting Hakai.

In PSF-treated lysates of HRMEC transfected with different amounts of Hakai protein, VEGF promoter luciferase activity (J), VEGF protein level (K) and grayscale analysis of VEGF expression were measured.

## Discussion

Pathologic neovascularization is characterized by proliferation and migration of abnormal cells [19, 20]. In this study, we showed that in vivo, PSF ameliorated RNV and corrected aberrant VEGF expression in an OIR mouse model and reduced IRNV in Vldlr − / − mice. In vitro, PSF reprogrammed the mitochondrial bioenergetic and glycolytic profiles under hypoxic conditions and inhibited hypoxia-induced angiogenesis in HRMECs, suggesting the participation of PSF in the proliferation and migration regulation in HRMECs during the development of pathological angiogenesis. In addition, we demonstrated the interaction between PSF and HIF-1α in HRMECs and Hakai recruitment to the inhibition of HIF-1α in a hypoxia-dependent manner, which may play a vital role in the regulation of their behavior responded to hypoxia. Thus, our data indicated that PSF is a potential therapeutic target in neovascularization-associated ophthalmopathy.

As the main producer of ROS in hypoxic cells, mitochondria determine the fate of cells under hypoxic conditions [21]. Our previous studies focused on the metabolic reprogramming in ocular diseases and found that the role of mir-451a/Atf2 in retinal pigment epithelium (RPE), Wnt signaling and PPARα in EPCs were realized through regulation of the metabolic cycle [22–24]. The role of HIF-1α in metabolic pathways can further help elucidate the effects of PSF on the bioenergetics pattern in HRMECs. Previous studies have reported that enhanced and stabilized HIF-1α regulates mitochondrial dynamics in hypoxia-induced pulmonary vascular remodeling and promotes ATP generation by enhancing anaerobic glycolysis [25–27]. For instance, HIF-1α induces cellular ROS production, inhibits mitochondrial respiration and electron transfer chain activity, while inducing glucose transporters, glycolytic enzymes, and apoptotic protein BNIP3 gene transcription, which increases the flow from glucose to lactic acid [28–30]. In the present study, we observed that PSF reprogrammed the mitochondrial bioenergetic and glycolytic profiles under hypoxic conditions and consequently inhibited angiogenesis in HRMECs.

We further observed that, under hypoxia stimulation, PSF bound to HIF-1α and inhibited VEGF gene transcription. These results indicated that PSF is a novel hypoxia-induced transcription factor that inhibits the expression of VEGF. Moreover, the relationship between PSF and HIF-1α must be carried out under hypoxia, which is consistent with our previous finding that PSF inhibits IgE and VEGF gene transcription in an IL-4- and IGF-1-dependent manner, respectively [9, 10]. We also demonstrated that an oxygen deficient environment elevated the levels of tyrosine phosphorylation in PSF. Therefore, we speculated that the phosphorylated status of PSF facilitates its interaction with other partner proteins such as Hakai, allowing it to play its diverse roles. For example, tyrosine phosphorylation induced by EGF or HGF treatment facilitated the recognition of the E-cadherin complex by Hakai, which eventually led to its proteolysis [31, 32]. In addition, the cytoplasmic relocalization of PSF induced by EGF treatment promotes the formation of a complex with breast tumor kinase, which leads to cell cycle arrest [33].

PSF can perceive dynamic changes in the organism or microenvironment through changes in the level of systemic or local cytokines, and then make accurate and targeted response to maintain homeostasis of the internal environment [34]. This finding has important clinical implications. Because the normal physiological VEGF levels maintain the normal structure and the function of blood vessels, over-inhibition of VEGF would cause a series of long-term complications [35]. Therefore, anti-VEGF treatment in clinic should ideally and selectively inhibit the pathologic VEGF but simultaneously maintain the physiologic VEGF level. Our findings indicate that PSF senses the changes in HIF-1α expression level to exactly distinguish between VEGF pathological and physiological levels. PSF can effectively restrain elevated pathological VEGF without affecting the normal function of the VEGF. Therefore, PSF may be a new target for specific inhibition of pathological VEGF expression.

Hakai, present in both the nucleus and the cytoplasm, was also found to participate in cell proliferation regulation [12, 13]. This ability of interacting with both E2 and the substrate facilitates Hakai to assume the responsibility for a substrate recognition module within ubiquitination system. Therefore, Hakai may coordinate and cooperate with several cellular activities and processes. It is reported that PSF is capable of regulating proliferation by recruiting E3 Ub ligases, it targets RNF43 and Hakai in colorectal cancer, leading to their ubiquitination and subsequent proteolytic cleavage [36]. Von Hippel–Lindau (Vhl) protein is a component of E3 Ub ligase, Under normoxic conditions, Vhl ubiquitinates HIF-1α, which leads to proteolytic destruction of HIF-1α through the Ub-proteasome pathway, which in turn contributes substantially to the inactivation of VEGF [37, 38]. However, hypoxia induces the expression of HIF-1α that activates VEGF gene transcription in the nucleus, which is involved in angiogenesis [4]. Based on the results of our study, we hypothesized that once HIF-1α enters the nucleus, PSF inhibits its expression by recruiting Hakai. Thus, the formation of the PSF-Hakai–HIF-1α junctional complex reduces the expression of VEGF and inhibits angiogenesis.

In summary, the results showed that PSF had an antagonistic effect on HIF-1α/VEGF signal transduction induced by hypoxia in vivo and in vitro. Moreover, PSF was involved in RNV regulation and could inhibit VEGF induced angiogenesis. To our knowledge, this is the first study to demonstrate that PSF functions as a repressor of HIF-1α activation through recruitment of the Hakai as well as by regulating mitochondrial oxidation and glycolytic function. These findings delineate a novel regulatory mechanism of HIF-1α/Hakai signaling, which may play a major role in the pathogenesis of neovascularization in ophthalmopathy.

## Conclusions

Pathological neovascularization can lead to eye disease and blindness in severe cases. Hypoxia is a major factor leading to neovascularization. In our study, we delineate a novel regulatory mechanism of HIF-1α/Hakai signaling, which may play a major role in the pathogenesis of neovascularization in ophthalmopathy. Ectopic PSF expression inhibited hypoxia-induced HIF-1α activation in the nucleus by recruiting Hakai to the PSF/HIF-1α complex, causing HIF-1α inhibition. Our results emphasize the relationship between the PSF-Hakai–HIF-1α signaling pathways, mitochondrial function, HRMEC proliferation and migration related to pathologic neovascularization. We concluded that PSF functions as a repressor of hypoxia-induced angiogenesis by promoting mitochondrial function. The significance of our study is to determine the role and related mechanism of PSF in the treatment of neovascular ophthalmopathy, and provide new ideas and targets for better treatment of pathological neovascular ophthalmopathy so as to improve the visual quality of patients.

## Materials and methods

### Animals

Vldlr − / − mice and wild type (WT) age-matched C57/BL6J mice were purchased from Jackson Laboratories (Bar Harbor, ME, USA). Care, use, and treatment of experimental animals were in agreement with the Association for Research in Vision and Ophthalmology (ARVO) Statement for the Use of Animals in Ophthalmic and Vision Research and approved by the Institutional Animal Care and Use Committee of Tianjin Medical University (Permit Number: SYXK 2009–0001).

### Oxygen induced retinopathy (OIR)

The induction process of OIR is as mentioned earlier. Briefly, C57/BL6J mice pups were housed in hyperoxia (75 ± 2% O_2_) environment during P7–P12 and followed by another 5 days’ room air (P12–P17), thereby causing retinal ischemia. OIR animals were allocated into three groups randomly: OIR, OIR + vehicle (OIR pups treated with rAAV vector), and OIR + PSF (OIR pups treated with rAAV–PSF). Construction, packaging, and intravitreal injection of rAAV were described previously. Age-matched C57/BL6J mice were used as controls and placed under constant normoxic conditions (P0–P17). Animals were sacrificed on P17 and their pupils were subjected to following experiments.

### Fundus fluorescein angiography (FFA)

As mentioned earlier, FFA was performed using the Micron III fundoscopy system (Phoenix Research Labs, Pleasanton, CA, USA). Simply put,, 5% sodium fluorescein was injected into the abdominal cavity of anesthetized animals with dilated pupils (Akorn, Decatur, IL, USA; 50 μl/mouse). The image is taken at a predetermined time (1, 3, 5 and 7 min). For Vldlr − / − mice, the number of fluorescent leakage points 3 min after injection was analyzed.

### Choroidal and retina flat mount

Vldlr − / − mice treated with rAAV–PSF by injecting 2 μl viral solution intravitreally on P12 were euthanatized on P17 with lethal doses of tribromoethanol (Avertin; Sigma, St. Louis, MO); their eyes were enucleated and fixed in 4% paraformaldehyde (PFA) for 1 h followed by gently rinsing them in PBS. The retina and eyecup, containing the RPE, choroid, and sclera, were dissected overnight at 4 °C, stained with Alexa Fluor 594 labeled Isoectin B4 (IB4 Alexa Fluor 594, 1:100 dilution; Molecular Probes, Eugene, OR, USA) in 1 × PBS containing 1 mM CaCl2 overnight, then washed in 1 × PBS for 30 min and flat-mounted separately in a mounting medium (Richard Allan Scientific, Kalamazoo, MI, USA) on slides. These images are captured by a fluorescence microscope. The total number of neovascularization was counted and the neovascularization area was measured by ImageJ software, and the treatment group was compared with the control group.

### Cell culture and transfection

The primary cultured HRMECs were bought from Cell Systems Corporation (Kirkland, WA, USA) and cultured in endothelial basal growth medium (Lonza, Walkersville, MD, USA) containing endothelial cell growth factor combination with 20% FBS and 50 U/mL endothelial cell growth factor (Lonza), and 1% insulin-transferrin-selenium. All the cells used in the experiment were cultured in 5%CO2 atmosphere at 37 °C. A hypoxic environment was realized by using a special incubator. Hypoxic cell culture conditions were culyured in 2% O_2_ atmosphere (by injecting N2). Cells were maintained under hypoxia for 3 h, then the culture medium was changed and the follow-up experiments were carried out under normal conditions. On the basis of manufacturer's specification, transient transfection was operated using Lipofectamine ®2000 reagent (Invitgen). siRNA for PSF and Hakai and siRNA for scrambling are bought from Ambion (Austin, Texas, USA). The details of VEGF- luciferase reporter gene construction (pGL3-VEGF) and PSF construction have been reported.

### Metabolic profile analysis

Analysis of metabolic spectrum by Seahorse XFe96 Flux Analyzer (Agilent, Santa Clara, CA, USA) by simultaneously quantifying OCR and extracellular acidification rate (ECAR). Cellswere separately transfected with PSF plasmid using lipofectamine 2000 transfection reagent (Invitrogen, USA). Specific compounds related to electron respiration chain, including oligomycin, FCCP, and RAAprepared in the cartridge, were sequentially added totrack the reaction of mitochontria; meanwhile, the reagent combination composed of glucose, oligomycin, and 2-DG were serially incorporated to evaluate the response of glycolysis process.

### Luciferase assay

HRMECs were inoculated in 12-wells plates at a density of 3 × 104/well, and were co-transfected with the plasmids containing pGL3-VEGF (0.5 μg), pRL-TK (0.5 μg), and PSF (0.5 μg) united with or without various doses of Hakai siRNA. Untransfected cells were viewed as control group. After 36 h incubation, cell culture lysis reagent (Promega) was used to collect cell lysate and luciferase activity was measured by a Dual Luciferase Reporter Assay System (Promega, Beijing, China) as previously described (14). Taking the renilla luciferase activity as the standard, the luciferase activity of firefly was normalized. The experiments were repeated three times.

### Co-immunoprecipitation

pCIneo-HIF-1α-HA and pcDNA3.1-His-PSF vectors were co-transfected into HRMECs. After 36 h incubation, the cells were exposed to hypoxic conditions for 3 h or left in normoxic condition as parallel control, and then suspended in RIPA lysis buffer. The whole of cell lysates were cultured with mouse monoclonal anti-HA (clone 16B 12; BabCO) or anti-His (Sigma) antibody, and rabbit polyclonal IgG antibody (Santa Cruz Biotechnology) was used as a contrast group, and then incubated with protein G/A-Sepharose (Amersham Biosciences). The binding protein was detected by SDS-PAGE and Western blotting with anti-HA antibody or anti-His antibody.

For endogenous binding assay HRMECs were exposed to hypoxia for 3 h or left mocked, and then suspended in RIPA lysis buffer. The total cell lysates were cultured with mouse monoclonal anti-PSF (Sigma), anti-HIF-1α (Upstate Biotechnology), or isotope-matched IgG antibody as mentioned above, and then incubated with protein G/A-Sepharose. Separation of immunoprecipitation proteins by SDS-PAGE and detection by blotting with anti-PSF or anti-HIF antibody (Zymed Laboratories Inc. Laboratories, CA, USA) or anti-Hakai (ab91185; Abcam) antibody were used.

### Cell viability

Determination of cell activity by cell counting kit-8 (Dojindo Laboratories, Kumamoto, Japan). Briefly, cells cultured in 96-well cell culture plate at 4 × 104 cells/well underwent specific intervention followed by incubating with blank DMEM basic medium (100 μL) containing 10% CCK-8 solution for 2 h at 37 °C incubator. The absorbance was measured at 450 nm on an Infinite 200 PRO Multimode Microplate Reader (Tecan Group Ltd., Switzerland).

### Hoechst staining

Hoechst staining was performed as reported previously. The cells were fixed 15 min in 4% formaldehyde at room temperature, washed with PBS, and stained 5 min with Hoechst 33,342 (100 μg / mL). The stained nuclei were watched under a fluorescence microscope (Olympus) with a 350-nm excitation filter to count the cell number.

### Tube formation assay

The formation of the measuring tube as mentioned earlier. Matrigel (BD Biosciences, Franklin Lakes, NJ, USA) was coated on 24-wells culture plates and polymerized for 30 min at 37 °C. HRMECs (1 × 105 cells) from each of the different treated groups were inoculated on the surface of matrix gel. The formation of the tube was photographed with a Zeiss digital camera and quantified by counting the number of connected cells in a randomly selected field and divided by the total number of cells in the same area.

### Wound migration assay

HRMECs cells were cultured in 12-wells plates with 4 × 10 ~ 5 cells per well. When a confluence degree of about 80% was reached, a linear wound was produced by scratching the cells with the tip of an aseptic straw. After injury, plates were washed with serum-free medium to get rid of floating cells and imaged using a phase contrast microscope before and after 12 h. In this assay, cell migration was measured by analyzing the number of cells that migrated into the scraped area.

### Transwell assay

A total of 1 × 105 cells/well were inoculated into a Transwell insert (Corning, Tewksbury, MA, USA) and then cultured in in serum-free medium. Add different stimuli to the lower chamber. The cells are then allowed to migrate through the polycarbonate filter at 37 °C for 24 h by scraping the non-migrated cells from the upper part of the filter. The migrating cells at the lower end of the filter were fixed with 4%PFA for 30 min, and then 20 min was stained with 0.1% crystal violet solution (Chinese Solarbio). The number of stained cells in 5 random fields per hole was counted by ImageJ software, and the average number of migrating cells was measured.

### Western blot analysis

The same amounts of the immunoprecipitated proteins were isolated on 10% SDS polyacrylamide gels, Separation of the same amount of immunoprecipitation proteins by 10% SDS polyacrylamide gels was done, followed by transfer to polyvinylidene fluoride (PVDF) membranes (Millipore, MA, USA) on a wet transfer apparatus (Bio-Rad) for 100 min at 90 V. The blocked membrane, treated with 5% nonfat milk, was subsequently incubated with the following specific primary antibodies: anti-phospho-tyrosine (#9411; Cell Signaling Technology), anti-PSF, or anti-Hakai (ab91185; Abcam) antibody with GAPDH as a reference at 4 °C overnight. After washing the membranes with TBST, the second antibody coupled with horseradish peroxidase (HRP) was incubated at room temperature for 2 h. Then, band detection, scanning, and band intensity analysis were detected using chemiluminescence kit (GE Healthcare, Freiburg, USA) and MultiSpectral Imaging System (EC3 410, UVP, Upland, CA, USA).

### Statistical analyses

All results were expressed as mean ± SD. All experiments were performed at least three times. Quantitative data analysis used two-tailed Student's t-test to compare the two groups, one-way or two-way analysis of variance (ANOVA) and Tukey's post hoc tests were used to compare three or more groups. The difference was statistically significant (P < 0.05).

## Data Availability

All data generated or analyzed during this research process is included in this published article.

## Abbreviations

PSF: Polypyrimidine tract-binding protein-associated splicing factor
HIF-1α: Hypoxia-inducible factor-1 alpha
VEGF: Vascular endothelial growth factor
IGF-1: Insulin-like growth factor 1
HGF: Hepatocyte growth factor
EGF: Epidermal growth factor
Ig: Immunoglobulin
HRMECs: Human retinal microcapillary endothelial cells
RNV: Retinal neovascularization
OIR: Oxygen-induced retinopathy
IRNV: Intra-retinal neovascularization
FFA: Fundus fluorescein angiography
PHD: Prolyl hydroxylase domain
pVHL: Produc von Hippel–Lindau gene

## References

[1] Wong BW, Marsch E, Treps L, Baes M, Carmeliet P (2017). Endothelial cell metabolism in health and disease: impact of hypoxia. EMBO J.

[2] Wu ZH, You F, Wen AY, Ma DY, Zhang PJ (2016). Physiological and morphological effects of severe hypoxia, hypoxia and hyperoxia in juvenile turbot (*Scophthalmus maximus* L.). Aquac Res.

[3] Luo L, Hong X, Diao B, Chen S, Hei M (2018). Sulfur dioxide attenuates hypoxia-induced pulmonary arteriolar remodeling via Dkk1/Wnt signaling pathway. Biomed Pharmacother.

[4] Zhang D, Lv FL, Wang GH (2018). Effects of HIF-1α on diabetic retinopathy angiogenesis and VEGF expression. Eur Rev Med Pharmacol Sci.

[5] Bahrami B, Shen W, Zhu L, Zhang T, Chang A, Gillies MC (2019). Effects of VEGF inhibitors on human retinal pigment epithelium under high glucose and hypoxia. Clin Exp Ophthalmol.

[6] Wang X, Wang G, Wang Y (2009). Intravitreous vascular endothelial growth factor and hypoxia-inducible factor 1a in patients with proliferative diabetic retinopathy. Am J Ophthalmol.

[7] Liu SC, Chuang SM, Hsu CJ, Tsai CH, Wang SW, Tang CH (2014). CTGF increases vascular endothelial growth factor-dependent angiogenesis in human synovial fibroblasts by increasing miR-210 expression. Cell Death Dis.

[8] Du SC, Zhu L, Wang YX, Liu J, Zhang D, Chen YL, Peng Q, Liu W, Liu B (2019). SENP1-mediated deSUMOylation of USP28 regulated HIF-1α accumulation and activation during hypoxia response. Cancer Cell Int.

[9] Dong L, Zhang X, Fu X, Zhang XZ, Gao XJ, Zhu MY, Wang XT, Yang ZX, Jensen ON, Saarikettu J (2011). PTB-associated splicing factor (PSF) functions as a repressor of STAT6-mediated Ig epsilon gene transcription by recruitment of HDAC1. J Biol Chem.

[10] Dong L, Nian H, Shao Y, Zhang Y, Li Q, Yi Y, Tian F, Li W, Zhang H, Zhang X (2015). PTB-associated splicing factor inhibits IGF-1-induced VEGF upregulation in a mouse model of oxygen-induced retinopathy. Cell Tissue Res.

[11] Castosa R, Martinez-Iglesias O, Roca-Lema D, Casas-Pais A, Díaz-Díaz A, Iglesias P, Santamarina I, Graña B, Calvo L, Valladares-Ayerbes M, Concha Á, Figueroa A (2018). Hakai overexpression effectively induces tumour progression and metastasis in vivo. Sci Rep.

[12] Liu Z, Wu Y, Tao Z, Ma L (2018). E3 ubiquitin ligase Hakai regulates cell growth and invasion, and increases the chemosensitivity to cisplatin in non small cell lung cancer cells. Int J Mol Med.

[13] Rodríguez-Rigueiro T, Valladares-Ayerbes M, Haz-Conde M (2011). Hakai reduces cell-substratum adhesion and increases epithelial cell invasion. BMC Cancer.

[14] Figueroa A, Fujita Y, Gorospe M (2009). Hacking RNA: Hakai promotes tumorigenesis by enhancing the RNA-binding function of PSF. Cell Cycle.

[15] Deep G, Gangar SC, Agarwal C, Agarwal R (2011). Role of E-cadherin in antimigratory and antiinvasive efficacy of silibinin in prostate cancer cells. Cancer Prev Res (Phila).

[16] Figueroa A, Kotani H, Toda Y, Mazan-Mamczarz K, Mueller EC, Otto A, Disch L, Norman M, Ramdasi RM, Keshtgar M, Gorospe M, Fujita Y (2009). Novel roles of hakai in cell proliferation and oncogenesis. Mol Biol Cell.

[17] Li HS, Zhou YN, Li L, Li SF, Long D, Chen XL, Zhang ZB, Feng L, Li YP (2019). HIF-1α protects against oxidative stress by directly targeting mitochondria. Redox Biol.

[18] Hu W, Jiang A, Liang J, Meng H, Chang B, Gao H, Qiao X (2008). Expression of VLDLR in the retina and evolution of subretinal neovascularization in the knockout mouse model's retinal angiomatous proliferation. Invest Ophthalmol Vis Sci.

[19] Hutchings G, Janowicz K, Moncrieff L, Dompe C, Strauss E, Kocherova L, Nawrocki MJ, Kruszyna Ł, Wąsiatycz G, Antosik P (2020). The proliferation and differentiation of adipose-derived stem cells in neovascularization and angiogenesis. Int J Mol.

[20] Yu X, Chen X, Zheng XD, Zhang ZT, Zhao XJ, Liu Y, Zhang HY, Zhang LX, Yu H, Zhang M (2018). Growth differentiation factor 11 promotes abnormal proliferation and angiogenesis of pulmonary artery endothelial cells. Hypertension.

[21] Fuhrmann DC, Brüne B (2017). Mitochondrial composition and function under the control of hypoxia. Redox Biol.

[22] Shao Y, Dong LJ, Takahashi Y, Chen JL, Liu X, Chen Q, Ma JX, Li XR (2019). miRNA-451a regulates RPE function through promoting mitochondrial function in proliferative diabetic retinopathy. Am J Physiol Endocrinol Metab.

[23] Shao Y, Chen J, Freeman W, Chen JL, Liu X, Chen Q, Ma JX, Li XR (2019). Canonical Wnt signaling promotes neovascularization through determination of endothelial progenitor cell fate via metabolic profile regulation. Stem Cells.

[24] Shao Y, Chen J, Dong LJ, He XM, Cheng R, Zhou KL, Liu JP, Qiu FF, Li XR, Ma JM (2019). A protective effect of PPARα in endothelial progenitor cells through regulating metabolism. Diabetes.

[25] Chen X, Yao JM, Fang X, Zhang C, Yang YS, Hu CP, Chen Q, Zhong GW (2019). Hypoxia promotes pulmonary vascular remodeling via HIF-1α to regulate mitochondrial dynamics. J Geriatric Cardiol.

[26] Wang Y, Han X, Fu M, Wang JF, Song Y, Liu Y, Zhang JJ, Zhou JM, Ge JB (2018). Qiliqiangxin attenuates hypoxia-induced injury in primary rat cardiac microvascular endothelial cells via promoting HIF-1α-dependent glycolysis. J Cell Mol Med.

[27] Lee YM, Kim GH, Park EJ, Oh TI, Lee SJ, Kan SY, Kang H, Kim BM, Kim JH, Lim JH (2019). Thymoquinone selectively kills hypoxic renal cancer cells by suppressing HIF-1α-mediated glycolysis. Int J Mol Sci.

[28] Chua YL, Dufour E, Dassa EP, Rustin P, Jacobs HT, Taylor CT, Hagen T (2010). Stabilization of hypoxia-inducible factor-1alpha protein in hypoxia occurs independently of mitochondrial reactive oxygen species production. J Biol Chem.

[29] Zhang Y, Liu D, Hu H, Zhang P, Xie R, Cui W (2019). HIF-1α/BNIP3 signaling pathway-induced-autophagy plays protective role during myocardial ischemia-reperfusion injury. Biomed Pharmacother.

[30] Yang L, Wu J, Xie P, Yu J, Li X, Wang J, Zheng H (2019). Sevoflurane postconditioning alleviates hypoxia-reoxygenation injury of cardiomyocytes by promoting mitochondrial autophagy through the HIF-1/BNIP3 signaling pathway. PeerJ.

[31] Perez-Moreno M, Jamora C, Fuchs E (2003). Sticky business: orchestrating cellular signals at adherens junctions. Cell.

[32] Wheelock MJ, Johnson KR (2003). Cadherin-mediated cellular signaling. Curr Opin Cell Biol.

[33] Lukong KE, Huot ME, Richard S (2009). BRK phosphorylates PSF promoting its cytoplasmic localization and cell cycle arrest. Cell Signal.

[34] Qi C, Guo R, Dong L, Zhang XM, Li XR (2015). Appfication of PSF in ophthalmology. Int Rev Ophthalmol.

[35] Tian F, Dong L, Ji J, Zhang Y, Li QT, Yi Y, Tian F, Li WB, Zhang H, Zhang XM (2016). Inhibition of PTB-associated splicing factor on IGF-1/VEGF signaling pathway in retinal vascular endothelial cells. China J Exp Ophthalmol.

[36] Miyamoto K, Sakurai H, Sugiura T (2010). Proteomic identification of a PSF/p54nrb heterodimer as RNF43 oncoprotein-interacting proteins. Proteomics.

[37] Buckley DL, Van Molle I, Gareiss PC, Tae HS, Michel J, Noblin DJ, Jorgensen WL, Ciulli A, Crews CM (2012). Targeting the von Hippel–Lindau E3 ubiquitin ligase using small molecules to disrupt the VHL/HIF-1α interaction. J Am Chem Soc.

[38] Jaakkola P, Mole DR, Tian YM, Wilson MI, Gielbert J, Gaskell SJ, Kriegsheim AV, Hebestreit HF, Mukherji M, Schofield CJ (2001). Targeting of HIF-α to the von Hippel–Lindau Ubiquitylation complex by O2-regulated prolyl hydroxylation. Science.

