# Correction: PSF functions as a repressor of hypoxia-induced angiogenesis by promoting mitochondrial function

**DOI:** 10.1186/s12964-023-01237-7

**Published:** 2023-07-24

**Authors:** Lijie Dong, Wenbo Li, Tingting Lin, Boshi Liu, Yaru Hong, Xiaomin Zhang, Xiaorong Li

**Affiliations:** 1 Tianjin Key Laboratory of Retinal Functions and Diseases, Tianjin, People’s Republic of China; 2 Tianjin International Joint Research and Development Centre of Ophthalmology and Vision Science, Tianjin, People’s Republic of China; 3 Eye Institute and School of Optometry, Tianjin Medical University Eye Hospital, 251 Fukang Road, Nankai, Tianjin, 300384 People’s Republic of China

**Correction: Cell Commun Signal 19, 14 (2021)**

**https://doi.org/10.1186/s12964-020-00684-w**

Following publication of the original article [1], the authors reported an error in Fig. 5b and c.

Due to an error in the manuscript preparation and proofreading stage, there are mistakes in Fig. 5b and c, including image misuse (Fig. 5b) and incorrect labeling (Fig. 5b and c). The figure presented in this correction article has been corrected.

**Fig. 5:**
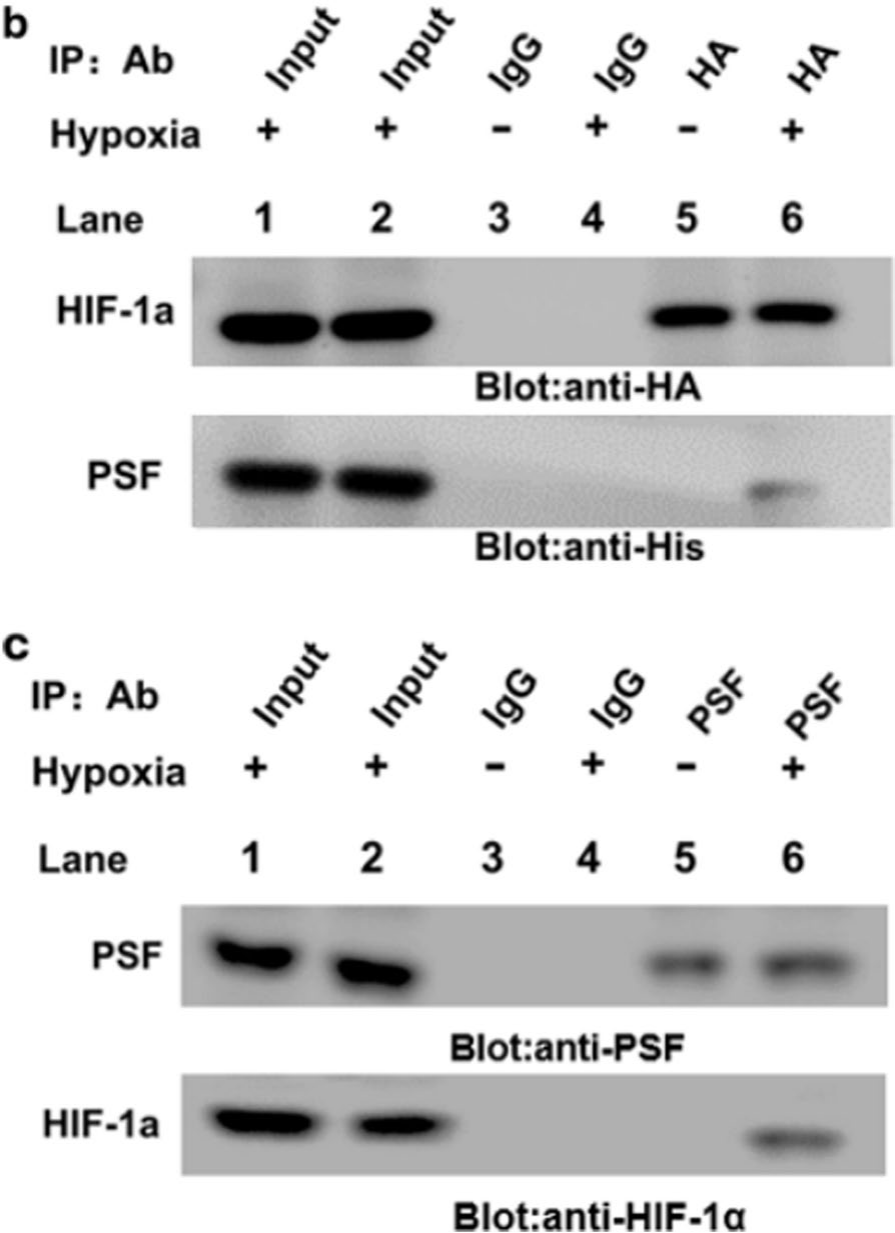
**b**-**c** PSF-HIF-1α complex formation under hypoxia induction. Ectopically expressed PSF interacted with HIF-1α were checked using anti-HA antibody in HRMECs as indicated (**a**) and anti-His antibody sedimentation (**b**). **c** Anti-HIF-1αor anti-PSF antibody-based immunoprecipitation with total cell lysates of HRMECs under hypoxia exposure was performed, followed by immunoblotting with the corresponding antibodies as indicated
